# Impact Position Estimation for Baseball Batting with a Force-Irrelevant Vibration Feature

**DOI:** 10.3390/s22041553

**Published:** 2022-02-17

**Authors:** Wei-Han Chen, Yang-Chih Feng, Ming-Chia Yeh, Hsi-Pin Ma, Chiang Liu, Cheng-Wen Wu

**Affiliations:** 1 Graduate Institute of Sports Equipment Technology, University of Taipei, Taipei 111036, Taiwan; gn01800083@gmail.com (W.-H.C.); yehmingchia@gmail.com (M.-C.Y.); 2 Department of Athletic Performance, National Taiwan Normal University, Taipei 106, Taiwan; 3 Department of Electrical Engineering, National Tsing Hua University, Hsinchu 300044, Taiwan; yfyfyfyfyfyf12321@gmail.com (Y.-C.F.); cww@ee.nthu.edu.tw (C.-W.W.); 4 Center for Sport Science and Technology, National Tsing Hua University, Hsinchu 300044, Taiwan; 5 National Baseball Research and Development Center, Taipei 111036, Taiwan

**Keywords:** sweet spot, node of vibration, bending modes of vibration, batting performance

## Abstract

In this work we propose a novel method for impact position estimation during baseball batting, which is independent of impact intensity, i.e., force-irrelevant. In our experiments, we mount a piezoelectric vibration sensor on the knob of a wooden bat to record: (1) 3600 vibration signals (waveforms) from ball–bat impacts in the static experiment—30 impacts from each of 40 positions (distributed 1–40 cm from the end of the barrel) and 3 intensities (drop heights at 75, 100, and 125 cm, resp.), and (2) 45 vibration signals from actual battings by three baseball players in the dynamic experiment. The results show that the peak amplitude of the signal in the time domain, and the peaks of the first, second, and third eigenfrequencies (EFs) of the bat all increase with the impact intensity. However, the ratios of peaks at these three EFs (1st/2nd, 2nd/3rd, and 1st/3rd) hardly change with the impact intensity, and the observation is consistent for both the static and dynamic experiments across all impact positions. In conclusion, we have observed that the ratios of peaks at the first three EFs are a force-irrelevant feature, which can be used to estimate the impact position in baseball batting.

## 1. Introduction

In baseball batting, the impact position of the ball on the bat is one of the key factors related to batting performance [1,2] and injury [3]. When the batter hits the ball at the sweet spot, it will result in minimum energy loss, maximum rebound energy, and the fastest ball exit speed [4]. Hitting at the sweet spot also leads to minimized impact vibration [5], and least vibration-induced pain or injury from a high ball–bat impact [3]. The sweet spot zone thus can be defined and verified by the vibration node (center of percussion or impact position) and coefficient of restitution [4,6,7]. In [8], the sweet spot is defined as a narrow impact zone, which is an area about two inches long and one-third of an inch wide along the bat, centered about six inches from the barrel end. However, in another work [5], the sweet spot of a wooden baseball bat is represented by an impact zone of only about 3 cm long, where the force and the impulse transmitted to the hands are both minimized [5].

Experiencing contact-induced feedback is necessary to learn how to utilize those feedback to develop better hitting performance [9,10,11,12]. Intuitively, the impact vibration as perceived by a human batter provides useful information to train the batter to hit the ball closer to the sweet spot [9]. Note that it is very difficult and unreliable to identify the impact position using the impact vibration feedbacks perceived by any particular batter. For that reason, some researchers have attempted to estimate the impact position in a more systematic way [13,14]. Fallon, Sherwood and Donaruma [13] evaluated different sensors, including the piezoelectric accelerometers, strain gauges, and microphones, trying to identify the best sensor for estimating the impact position. In their static tests, the piezoelectric sensors presented the best result in estimating the impact position, with an average absolute error of 4 cm—being able to estimate the position within 5 cm and 2.5 cm, 63% and 38% of the time, respectively. However, their method requires installing four accelerometers on the bat, including both ends, mid-point, and handle of the bat, to synchronize all sensors for calculating the relative trigger time [13]. Recently, Osawa, Tanaka, Yanai and Sano [14] used the vibration signal retrieved from polyvinylidene difluoride (PVDF) films on the knob of the bat, and estimated the impact positions by calculating the peak amplitude of the filtered signal within the 80–300 Hz band. In their static experiment, they collected five measurement data (i.e., 5 impacts) from each of the nine positions (distributed 1–40 cm from the end of the barrel, with a stride of 5 cm) and two intensities (drop heights at 1 and 1.5 m, respectively). Their results show that the average root-mean-square error for the impact position estimation was 7.9 cm. However, the peak amplitude is dependent on the impact intensity (i.e., the ball and/or bat-swing velocity), which introduces unpredictable error, so it is difficult to accurately estimate the impact position in real play or batting practice.

Note that the impact intensity is influenced by the ball and/or bat-swing velocity in real batting [10]. A pitcher usually tries to confuse the batter’s timing by random ball-types with different velocities, to prevent the batter from making good contact. Common ball types include the fastball, curveball, slider, cutter, sinker, splitter, change-up, etc., which have different velocities [15] and trajectories [16]. The pitches with random velocities and trajectories can cause deviation of the impact position for a typical batter. It is crucial to identify the deviation in order to help the players to improve their batting performance. Apparently, we need an accurate impact position estimation approach that is force-irrelevant (i.e., independent of the impact intensity). Moreover, it is necessary to check the feasibility of the specific features in the vibration signal under not just the controlled experiments with fixed impact intensity, but also real batting experiments with random impact intensity. 

We hypothesize that, if the strength of the vibration signals can be normalized in some way, e.g., by calculating certain ratios of the raw signal features, we may come up with a force-irrelevant feature that is not affected by the impact intensity, i.e., the normalized (ratioed) vibration signal feature is independent of the impact intensity, but is dependent on the impact position. Regarding the force-irrelevant features proposed in this study for impact position estimation, please refer to the Methods (Section 2.1) for more information.

Previous studies analyze the signal in the time domain [13,14], which has only one peak representing the highest amplitude of the vibration signal at the ball–bat contact. In this work, in addition to the original time domain signal, we also analyze the frequency domain signals. Through the spectrum analysis, it has been shown that a baseball bat has more than three bending modes of vibration, which occur at different eigenfrequencies (EFs) and have different magnitudes [4]. Therefore, in this paper, we investigate the effect of impact intensity on the strength-related features of the vibration signal, and verify the consistency of the effect in the features, under the controlled static experiments with known impact intensity, as well as the dynamic (real batting) experiments with random impact intensity. The vibration signal features taken are the peak amplitude of the time-domain signal, the peaks at the first three EFs in frequency domain, as well as the ratios of peaks at first three EFs.

We perform a series of static and dynamic experiments, and show that the ratios of peaks at the first three EFs in the frequency domain are a force-irrelevant feature, which can be used to estimate the impact position in baseball batting.

## 2. Materials and Methods

### 2.1. Proposed Force-Irrelevant Features for Impact Position Estimation

Previous studies analyze the signal in the time domain [13,14], and the peak (the highest amplitude) of the vibration signal at the ball–bat contact shows a significant contribution to the estimation of the impact location. When the node of vibration mode is used as the definition of the sweet spot, the vibration generated when the ball hits the sweet spot will be minimal, while the vibration generated by the hitting position far away from the sweet spot will be larger. Therefore, the peak amplitude might be a simple method to estimate the impact location of a bat in the time domain signal.

Assume the original time domain vibration signal after one batting is denoted as x[d,i], where d is the impact position starting from the end of the barrel, i is the time index from 0 to n−1, and n is the total length of one batting process. The peak in the primary bending mode can be calculated by the following steps:

1. Send x[d,i] to a bandpass filter (80 Hz–400 Hz) to get the primary bending mode signal $\hat{x}\left[d,i\right]$.
2. Get the peak magnitude P[d] in the primary bending mode by $P\left[d\right]=\max\left(\hat{x}\left[d,i\right]\right)$.

However, the value of peak magnitude P[d] depends on the impact intensity. We can find other features to remove the intensity dependency. In addition to the original time domain signal, we can also analyze the frequency domain signals. The peak magnitude of the first three eigenfrequencies can be calculated as follows:

1. Send x[d,i] to three bandpass filters (80 Hz–280 Hz, 400 Hz–600 Hz, 880 Hz–1080 Hz) to get the first three bending mode signals $\hat{x_{j}}\left[d,i\right]$, where *d* is the impact position starting from grip, i=0,⋯,n−1, and j=1,2,3.
2. Apply fast Fourier transform to $\hat{x_{j}}\left[d,i\right]$ and get the frequency-domain data $\hat{X_{j}}\left[d,f\right]$.
3. Process $\hat{X_{j}}\left[d,f\right]$ to get the peak magnitude $M_{j}\left[d\right]$ in the three eigenfrequencies by $M_{j}\left[d\right]=\max\left(ℱ\left(\hat{X_{j}}\left[d,f\right]\right)\right)$.

The magnitudes $M_{j}\left[d\right]$ of various bending modes have different shape relationships with the impact position [17]. It is recognized that the *sweet spot* zone can be framed by nodes of the primary bending mode and the second bending mode [4,5,17,18,19,20,21]. Because of the characteristic of natural frequency, no matter where the impact position is located, under the same external conditions, the eigenfrequency will not change, only the peak magnitude of the spectrum will change accordingly. Assume the signal strengths in all *bending modes* or *EFs* are equally affected by the impact intensity in a similar proportion, we should be able to remove the effect of impact intensity by taking the ratios. Therefore, $\frac{M_{1}\left[d\right]}{M_{2}\left[d\right]}$, $\frac{M_{2}\left[d\right]}{M_{3}\left[d\right]}$, and $\frac{M_{1}\left[d\right]}{M_{3}\left[d\right]}$ can be selected as the proposed force-irrelevant features for the impact position estimation.

### 2.2. Study Design

We mount a piezoelectric vibration sensor on the knob of a wooden bat to record: (1) 3600 vibration signals (waveforms) from ball–bat impacts in the static experiment with known impact intensity—30 impacts from each of 40 positions (distributed 1–40 cm from the end of the barrel) and 3 intensities (drop heights at 75, 100, and 125 cm, resp.), and (2) 45 vibration signals from actual battings by three baseball players in the dynamic experiment with random impact intensity and impact position. The features of the vibration signal are taken as the peak amplitude of the time-domain signal, the peaks at the first three EFs in the frequency domain, and three ratios of peaks at the first three EFs. We verify the effects of impact intensity on those vibration signal features, and the consistency of the features under static and dynamic experiments. Informed consent is obtained from participants after detailed experiment protocol and risks associated with this study are provided. All procedures performed were approved by the Institutional Review Board and in accordance with the Helsinki declaration.

### 2.3. Vibration Sensor

**Figure 1:**
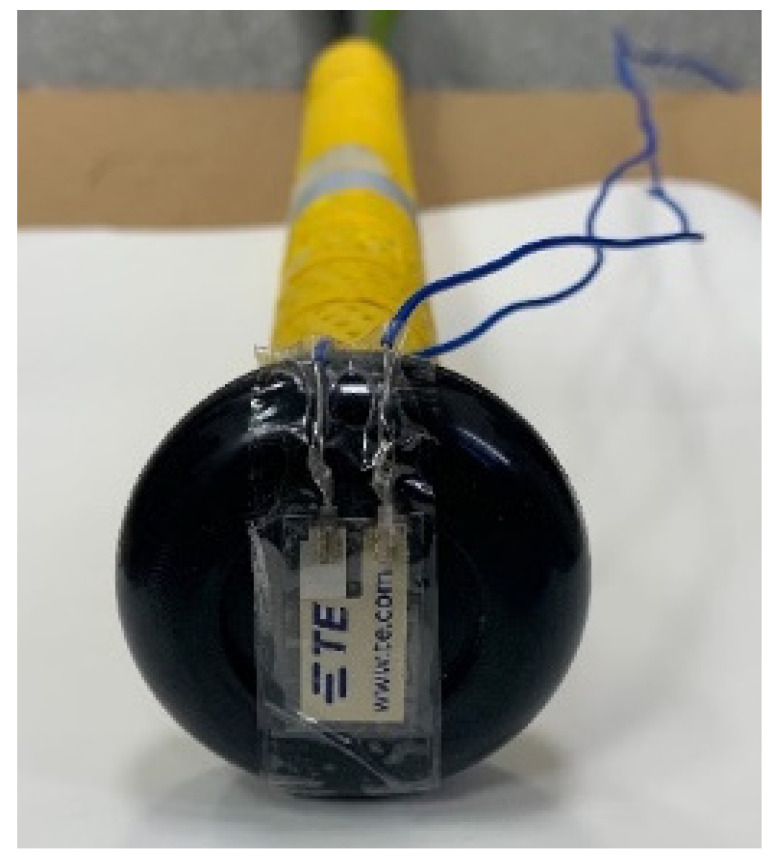
The LDT0-028K sensor mounting position. The longer side of this sensor is parallel to the impact direction.

We use a PVDF sensor (LDT0-028K, TE connectivity, Schaffhausen, Switzerland) to record the vibration signals from ball–bat impacts. This sensor is mounted at the knob of the bat (Figure 1) and the longer side of the sensor is parallel to the impact direction. The LDT0-028K is composed of a 28 µm thickness piezoelectric PVDF polymer film and a silver ink electrode, which are laminated to a polyester substrate to a thickness of 125 µm. The LDT0-028K is also a flexible piezoelectric sensor that can be well operated at environmental temperature in the range 0~85 °C, and generates voltage endogenously during deformation or stress without additional power supply.

### 2.4. Static Experiment

#### 2.4.1. Data Collection

In the static experiment, a total of 3600 vibration signals from ball–bat impacts are recorded to obtain a dataset with 30 impacts from 40 positions (distributed 1–40 cm from the end of the barrel) and 3 intensities (an official baseball drop heights at 75, 100, and 125 cm, resp.). Each signal contains 20,000 data points in 3.2 s with a sampling frequency of 6250 Hz. The characteristics of the wooden bat (J143M, Old hickory bat company, Goodlettsville, TN, USA) used is 936.15 g in mass, 85.3 cm in length, 29.7 cm in centroid from the end of the barrel, and 6.435 cm in maximum diameter. 

#### 2.4.2. Experimental Apparatus

**Figure 2:**
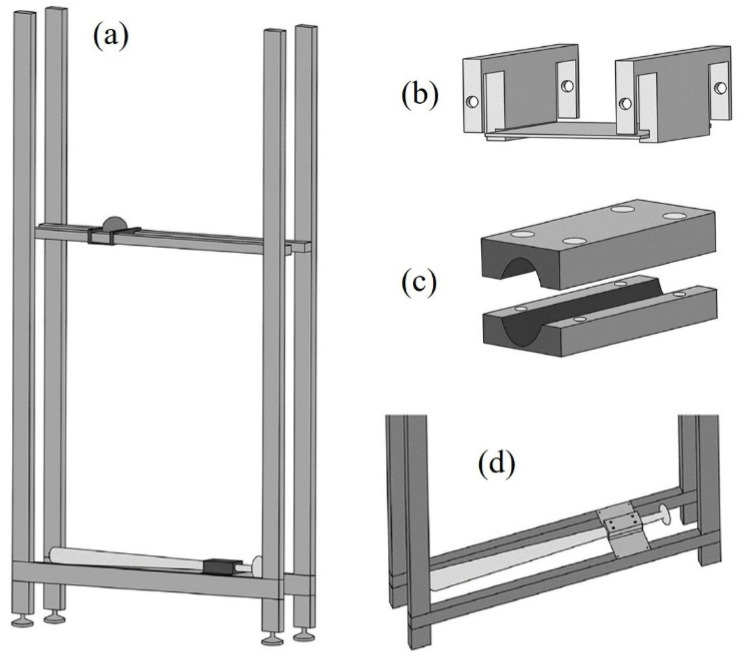
The experimental apparatus (**a**). The height of the upper horizontal aluminum extrusion can be adjusted to change the drop height of the baseball. The holder (**b**) placed on upper horizontal aluminum extrusion can be moved horizontally, which is used to fix the drop position of baseball. The gripper (**c**) placed on lower aluminum extrusion (**d**) is used to clamp the bat.

We customize an apparatus to control the horizontal and vertical drop positions of the baseball (Figure 2). The material of the horizontal and vertical bars in this apparatus is an aluminum extrusion. The adjustable height of upper horizontal aluminum extrusion allows changing the drop height of the ball. Two auxiliary tools (holder and gripper) made by a 3D printer using the acrylonitrile butadiene styrene are used to fix the ball and the bat, respectively. The holder (Figure 2b) placed on the upper horizontal aluminum extrusion can move horizontally to adjust the drop position of the baseball, while the gripper (Figure 2c) is placed at the lower aluminum extrusion of the apparatus to clamp the bat (Figure 2d).

### 2.5. Dynamic Experiment

**Figure 3:**
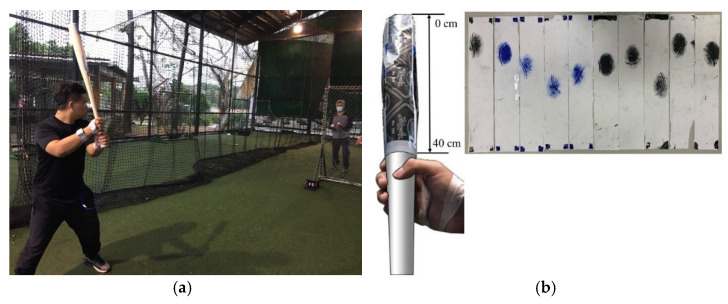
Soft toss batting is performed by a trained personnel in the dynamic experiment (**a**) and uses the wrapped carbon copy paper to mark the impact positions (**b**).

In the dynamic experiment, a total of 45 vibration signals from actual battings by three male baseball players are recorded with 15 battings performed by each subject. A trained personnel performs *soft toss batting* with ~5 m of toss distance from the participant who performs batting singly (Figure 3a). All players are instructed to hit the ball tossed with random swing speed and bat position. The participants are right-handed with more than six years of baseball training experience, including a coach (age: 57 years, height: 175 cm, mass: 82 kg) and two college baseball players (Player-A, age: 25 years, height: 168 cm, mass: 90 kg; Player-B, age: 23 years, height: 175 cm, mass: 78 kg). A wooden bat and 15 official game baseballs same as in the static experiment are used in the dynamic experiment. The vibration signals from each ball–bat impact are collected and analyzed in both static and dynamic experiments. The contact spot of each bat-ball impact is marked by a carbon copy paper attached to the bat (Figure 3b). We then identify the impact position as the center of the contact marked on this paper relative to the longitudinal axis from the end of the barrel (1–40 cm).

### 2.6. Vibration Signal Features

#### 2.6.1. Peak Amplitude in Time Domain

The primary, second and third bending modes of vibration via the wood bat occur at about 180, 500, and 980 Hz, respectively. We use a bandpass filter in the range 80–400 Hz to extract the vibration signal of primary bending mode. We then calculate the absolute value of the largest peak (P[d]) from the filtered output signal, which is considered a parameter that represents the intensity of the primary bending mode of vibration [14].

#### 2.6.2. Peaks at the Eigenfrequencies in Frequency Domain

The frequencies in the first three vibration modes represent the first, second, and third EFs. We apply Fourier transform to convert the vibration signals to power spectral density to extract the peaks (magnitudes) of the different eigenfrequencies. Thereafter, we take the first, second, and third eigenfrequencies as the center, extracted the bandwidth of 200 Hz, respectively (80 Hz–280 Hz, 400 Hz–600 Hz, 880 Hz–1080 Hz), to obtain the peak at the first eigenfrequency ($M_{1}\left[d\right]$), peak at the second eigenfrequency ($M_{2}\left[d\right]$), and peak at the third eigenfrequency ($M_{3}\left[d\right]$) from these frequency bands.

#### 2.6.3. Ratio of the Peaks at the Eigenfrequencies

We calculate three ratios of $M_{j}\left[d\right]$: (1) ratio of $M_{1}\left[d\right]$ to $M_{2}\left[d\right]$ ($\frac{M_{1}\left[d\right]}{M_{2}\left[d\right]}$), (2) ratio of $M_{2}\left[d\right]$ to $M_{3}\left[d\right]$ ($\frac{M_{2}\left[d\right]}{M_{3}\left[d\right]}$), and (3) ratio of $M_{1}\left[d\right]$ to $M_{3}\left[d\right]$ ($\frac{M_{1}\left[d\right]}{M_{3}\left[d\right]}$).

### 2.7. Statistical Analysis

SPSS 23 software for Windows (IBM Corp., Armonk, NY, USA) is applied for data analysis. One-way analysis of variance is used to assess the difference among three impact intensities (drop height: 75, 100, and 125 cm) in all signal features at each impact position. Intraclass correlation coefficients (ICC) using two-way mixed single measures with absolute agreement are employed to assess the consistency of all signal features between the static experiment and the dynamic experiment across different impact positions. For the ICC analysis, because there were 90 data (3 intensity × 30 trials) at each impact position in the static experimental, the average is taken to correspond to the similar impact position (to the nearest whole number) in the dynamic experiment. Strength of agreement is based on the following thresholds: ICC = 0–0.5 (poor), ICC = 0.5–0.75 (moderate), ICC = 0.75–0.9 (good), and ICC > 0.9 (excellent) [22]. The level of significance is set at *p* < 0.05.

## 3. Results

**Figure 4:**
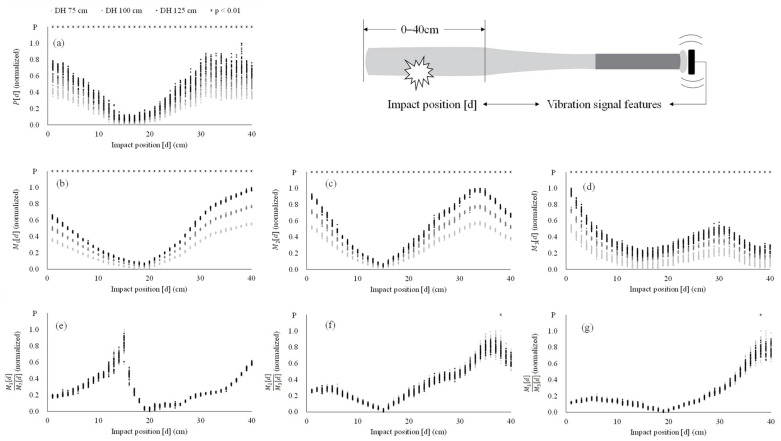
The scatter plot of strength of vibration signal features and impact position under different impact intensities in the static experiment. The drop height (DH) of the ball is 75 (light gray dots), 100 (dark gray dots), and 125 cm (black dots) to result in three impact intensities. All amplitudes are normalized by min-max normalization (=[X − X_min_]/[X_max_ − X_min_]). * = a significant difference among three impact intensities (*p* < 0.01). P[d] (**a**) = absolute largest peak in time domain; $M_{1}\left[d\right]$ (**b**) = peak at the first eigenfrequency; $M_{2}\left[d\right]$ (**c**) = peak at the second eigenfrequency; $M_{3}\left[d\right]$ (**d**) = peak at the third eigenfrequency; $\frac{M_{1}\left[d\right]}{M_{2}\left[d\right]}$ (**e**) = ratio of $M_{1}\left[d\right]$ to $M_{2}\left[d\right]$; $\frac{M_{2}\left[d\right]}{M_{3}\left[d\right]}$ (**f**) = ratio of $M_{2}\left[d\right]$ to $M_{3}\left[d\right]$; $\frac{M_{1}\left[d\right]}{M_{3}\left[d\right]}$ (**g**) = ratio of $M_{1}\left[d\right]$ to $M_{3}\left[d\right]$.

**Figure 5:**
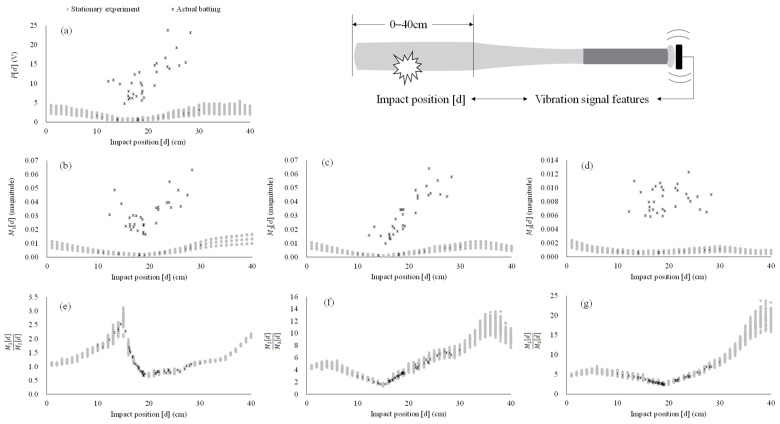
The scatter plot of strength of vibration signal features and impact positions in the static experiment (grey dots) and dynamic experiment (black crosses). P[d] (**a**) = absolute largest peak in time domain; $M_{1}\left[d\right]$ (**b**) = peak at the first eigenfrequency; $M_{2}\left[d\right]$ (**c**) = peak at the second eigenfrequency; $M_{3}\left[d\right]$ (**d**) = peak at the third eigenfrequency; $\frac{M_{1}\left[d\right]}{M_{2}\left[d\right]}$ (**e**) = ratio of $M_{1}\left[d\right]$ to $M_{2}\left[d\right]$; $\frac{M_{2}\left[d\right]}{M_{3}\left[d\right]}$ (**f**) = ratio of $M_{2}\left[d\right]$ to $M_{3}\left[d\right]$; $\frac{M_{1}\left[d\right]}{M_{3}\left[d\right]}$ (**g**) = ratio of $M_{1}\left[d\right]$ to $M_{3}\left[d\right]$.

The result of one-way analysis of variance reveals a significant increase with impact intensities (*p* < 0.001, Figure 4) in all features except for the ratios of $M_{j}\left[d\right]$. In the ratios of $M_{j}\left[d\right]$, the significant difference among different impact intensities only exists in $\frac{M_{2}\left[d\right]}{M_{3}\left[d\right]}$ (*p* = 0.008) and $\frac{M_{1}\left[d\right]}{M_{3}\left[d\right]}$ (*p* = 0.005) with impact position at 38 cm. Figure 5 represents the scatter plot of the strength of vibration signal features and impact position under static experiment and dynamic experiment. The impact positions in the dynamic experiment range from 10 to 30 cm. The consistency between static experiment and dynamic experiment across impact positions (10–30 cm) are poor in P[d] (ICC = 0.024, *p* = 0.354), $M_{1}\left[d\right]$ (ICC = 0.017, *p* = 0.388), $M_{2}\left[d\right]$ (ICC = 0.042, *p* = 0.272), and $M_{3}\left[d\right]$ (ICC = −0.001, *p* = 0.511), while the consistency of $\frac{M_{1}\left[d\right]}{M_{2}\left[d\right]}$ (ICC = 0.980, *p* < 0.001), $\frac{M_{2}\left[d\right]}{M_{3}\left[d\right]}$ (ICC = 0.991, *p* < 0.001), and $\frac{M_{1}\left[d\right]}{M_{3}\left[d\right]}$ (ICC = 0.992, *p* < 0.001) are excellent between static experiment and dynamic experiment across impact positions.

## 4. Discussion

In this work, we propose a novel feature of the vibration signal via batting (i.e., ratios of $M_{j}\left[d\right]$) and verify the effect of impact intensity on the features, as well as the consistency of features under the static experiment (controlled) and the dynamic (real batting) experiment. Our results demonstrate the ratios of $M_{j}\left[d\right]$ (i.e., $\frac{M_{1}\left[d\right]}{M_{2}\left[d\right]}$, $\frac{M_{2}\left[d\right]}{M_{3}\left[d\right]}$, and $\frac{M_{1}\left[d\right]}{M_{3}\left[d\right]}$) of the bat hardly changes with the impact intensity (Figure 4e–g). This observation is consistent in both static and dynamic experiments across all impact positions (ICC > 0.98, *p* < 0.001, Figure 5e–g). As expected, the P[d], $M_{1}\left[d\right]$, $M_{2}\left[d\right]$, and $M_{3}\left[d\right]$ all increase in accordance with the change of impact intensity (Figure 4a–d) in the static and the dynamic experiments (Figure 5a–d, ICC < 0.10, *p* > 0.05). Since the proportions of $M_{1}\left[d\right]$, $M_{2}\left[d\right]$, and $M_{3}\left[d\right]$ increase with impact intensity are similar, the effect of impact intensity can be removed by calculating the ratios of $M_{j}\left[d\right]$. As a result, the ratios of $M_{j}\left[d\right]$ are a force-irrelevant feature, i.e., independent of impact intensity, which can be used to estimate the impact position in baseball batting.

Along with previous work [14], we observe peak amplitude of the vibration signal in time domain is increased by the impact intensity resulting in a v-shaped relationship with impact position (Figure 4a). Moreover, we have also found that the P[d] on different impact positions increase accordingly with the change of impact intensity (Figure 4b–d). The distributions between the strength of the features and impact positions in $M_{1}\left[d\right]$, $M_{2}\left[d\right]$, and $M_{3}\left[d\right]$ similize the shape of V, N, and W, respectively (Figure 4b–d). The nodes of these three bending modes of vibration fall within the range of 15–19 cm from end of the barrel, which is comparable to previous findings [2,4,5,8,18]. However, note that these strength-related features (i.e., P[d] and $M_{j}\left[d\right]$) are dependent on the impact intensity (i.e., the ball and/or bat-swing velocity), which introduces unpredictable error, and further makes it difficult to accurately estimate the impact position in real play or batting practice due to the varied velocity of pitching [15,23] and swing [24]. This phenomenon is verified in our dynamic experiment. Our result reveals that P[d] and $M_{j}\left[d\right]$ for both static experiment and dynamic experiment are inconsistent (ICC < 0.10, *p* > 0.05) with very large deviation. Figure 5 shows that most of distribution (black crosses) in these strength-related features in the dynamic experiment are substantially deviated from those of distributed area (grey dots) from the static experiment. Therefore, it is critical to establish an novel feature to capture force-irrelevant (i.e., independent of the impact intensity) vibration signal for accurate impact position estimation. 

As proposed, the strength of the vibration signals can be normalized by calculating the ratios of the signal features. In doing so, a force-irrelevant feature is established, which is not affected by the impact intensity while remaining dependent on the impact position. In this paper, we demonstrate that the calculation of $M_{j}\left[d\right]$ ratios effectively eliminates the effect of impact intensity (Figure 4). The ratios of $M_{j}\left[d\right]$ hardly change with the impact intensity and are consistent whether in the static experiment (controlled) with known impact intensity or the dynamic experiment (real batting) with random impact intensity across all impact positions (Figure 4e–g and Figure 5e–g). With the ratios of $M_{j}\left[d\right]$ being a better feature of the vibration signal in comparison with strength-related features taken to estimate the impact position, plus its feasibility to apply in real settings, we suggest that researchers can use the ratios of $M_{j}\left[d\right]$ to build an algorithm for predicting the ball–bat impact position.

Figure 5e–g presents a complex non-linear relationship between the three ratios of $M_{j}\left[d\right]$ and the impact position, thus a linear regression may be unsuitable to establish the best predictive model. It may be feasible to build an accurate prediction model, namely, several segmented linear or non-linear relationships between the ratios of $M_{j}\left[d\right]$ and the impact position. For example, a simple linear regression can be built via $\frac{M_{1}\left[d\right]}{M_{2}\left[d\right]}$ across impact position ranged from 0 to 15 cm (Figure 5e) for estimating this impact position zone, while $\frac{M_{2}\left[d\right]}{M_{3}\left[d\right]}$ (Figure 5f) and $\frac{M_{1}\left[d\right]}{M_{3}\left[d\right]}$ (Figure 5g) can be used to attain two segmented regressions for estimating impact position zones varied from 16 to 25 cm and 26 to 40 cm, respectively. However, the prediction model requires a decision criterion to determine which feature and regression equation to use. Due to the essence of different distribution shapes from impact positions of the three ratios of $M_{j}\left[d\right]$, a high-accuracy prediction model may be achieved through advanced approaches, such as decision tree regressor or deep machine learning [25,26]. Further study is warranted to investigate this presupposition.

There are some limitations of this study. First, this work only operates with a wooden bat, limiting the fact that different materials of the bat may result in different vibration characteristics [18,21] and batting performance [21,27,28]. Moreover, in real play, a batter usually encounters not only different pitch velocities, but also various pitch types (fastball, curveball, slider, cutter, change-up) [15] and trajectories [16]. Further work is encouraged to verify the assumption of this study under different pitching and batting conditions. Another limitation of this study is that we used a wired vibration sensor to ensure a high sampling frequency and measurement range; however, it might not be convenient to apply in the field under a wired setting. Currently, wireless inertial sensors (inertial measurement units) have been applied for measurements (e.g., bat speed, time-to-impact, and attack angle, etc.) in baseball batting via wearable devices, e.g., Garmin Impact™ Bat Swing Sensor. These sensors are installed on the knob of the bat and can also measure vibration (acceleration) signal from batting, which is similar to our experimental setup. If the *M_j [d]* ratios obtained by using wireless inertial sensors are as feasible as the finding in the present study, utilizing wearable products for measuring batting positions in field is expected with further investigation.

In conclusion, the vibration signal via baseball bat is a critical parameter for estimating the impact position; however, it is not only related to the impact position, also greatly affected by the impact intensity. To make a breakthrough in impact position estimation, i.e., unaffected by impact intensity, we propose using $M_{j}\left[d\right]$ ratios as new vibration signal features. We conclude that this new $M_{j}\left[d\right]$ ratios are force-irrelevant features associated with the impact position, which can be further employed to estimate the impact position in baseball batting. Further work is needed to demonstrate whether the ratio of $M_{j}\left[d\right]$ is universally feasible in different bat materials, batting conditions, and measurement sensors (e.g., wireless inertial measurement units), to implement valid predictive algorithms, and to clarify the estimated accuracy of impact position in actual batting.

## Data Availability

The data presented in this study are available on request from the corresponding author.

## Abbreviations

EFs	eigenfrequencies
ICC	intraclass correlation coefficients
PVDF	polyvinylidene difluoride
P[d]	absolute largest peak in time domain
$M_{1}\left[d\right]$	peak at the first eigenfrequency
$M_{2}\left[d\right]$	peak at the second eigenfrequency
$M_{3}\left[d\right]$	peak at the third eigenfrequency
$\frac{M_{1}\left[d\right]}{M_{2}\left[d\right]}$	$\mathrm{ratio}\;\mathrm{of}\;M_{1}\left[d\right]$ $\;\mathrm{to}\;M_{2}\left[d\right]$
$\frac{M_{2}\left[d\right]}{M_{3}\left[d\right]}$	$\mathrm{ratio}\;\mathrm{of}\;M_{2}\left[d\right]$ $\;\mathrm{to}\;M_{3}\left[d\right]$
$\frac{M_{1}\left[d\right]}{M_{3}\left[d\right]}$	$\mathrm{ratio}\;\mathrm{of}\;M_{1}\left[d\right]$ $\;\mathrm{to}\;M_{3}\left[d\right]$
